# Involvement of NRF2 and AMPK signaling in aging and progeria: a digest

**DOI:** 10.1016/j.redox.2025.103782

**Published:** 2025-07-21

**Authors:** Eleni Petsouki, Vasileios Gerakopoulos, Despoina D. Gianniou, Elke H. Heiss, Ioannis P. Trougakos

**Affiliations:** a Department of Pharmaceutical Sciences, Division of Pharmacognosy, University of Vienna, Faculty of Life Sciences, Josef-Holaubek-Platz 2, 1090, Vienna, Austria; b Department of General Surgery, Division of Visceral Surgery, Medical University of Vienna, Waehringer Guertel 18-20, A-1090, Vienna, Austria; c Department of Cell Biology and Biophysics, Faculty of Biology, National and Kapodistrian University of Athens, Athens, 15784, Greece

**Keywords:** Aging, Progeria, AMPK, NRF2

## Abstract

Aging refers to a *gradual, continuous process of natural change* which is accompanied by progressive loss in physiological functions and an increased risk of frailty, disease, and death. Cells face a declining capacity to adapt homeostasis after perturbation, resulting among others in an imbalance in reactive species production and damage removal, as well as in energy or nutrient sensing and usage. NRF2 (Nuclear factor E2 p45‐related factor 2) is a transcription factor primarily known to regulate the expression of genes involved in cellular defense against oxidative, proteotoxic, or xenobiotic stress. AMPK (AMP-activated protein kinase), a serine/threonine kinase, serves as a central sensor of cellular energy status, maintaining ATP levels by tweaking the ratio of anabolic and catabolic pathways. Cooperativity between AMPK and NRF2 signaling, which goes beyond mere parallel activation in situations of cellular stress, has been previously described. This narrative short review zooms in the current understanding of NRF2 and AMPK signaling, alone or in concert, in aging and Hutchinson–Gilford Progeria Syndrome (HGPS), a genetic disorder characterized by premature aging.

## 1 Introduction

### 1.1 Aging and progeria

Aging is a biological process influenced by genetic and biological mechanisms that are closely connected to lifespan and play a central role in the development of diseases [1]. Various hallmarks of aging have been defined, including telomere attrition, cellular senescence, genome instability, mitochondrial impairment, inflammation, a decreased stem cell pool and regenerative capacity, disrupted proteostasis and epigenetic alterations [2]. Aging is the single most important risk factor for cardiovascular diseases [[3], [4], [5]], neurodegenerative diseases [[6], [7], [8]], musculoskeletal disorders [9], cancer [[10], [11], [12], [13]] and immune system disorders [10,[14], [15], [16]]. Consequently, the considerably increasing lifespan since the mid of the twentieth century is not reflected by an equal increase in health span (i.e. time of life free from chronic disease and disability), creating a physical, psychological and financial burden for the affected people, their relatives and society [[17], [18], [19]]. Understanding the mechanisms of aging might enable adequate interventions to relieve the strains associated with aged populations.

Over time, several theories evolved to explain aging. For instance, the initial “Free Radical Theory of Aging” proposed that any produced reactive oxygen species (ROS) infer damage onto cells and primarily drive aging [20]. This theory has meanwhile been proven to be oversimplistic, as numerous studies have challenged its exclusivity and general validity. The observation that impaired mitochondrial function can lead to both energy deficits and the accumulation of harmful reactive species gave rise to the “Mitochondrial Dysfunction Theory of Aging” [21,22]. Paradoxically, there is also evidence that inhibiting mitochondrial respiration can extend lifespan [23,24]. Resulting in low levels of ROS, it can improve systemic defense mechanisms by inducing an adaptive response that allows better handling of incoming stressors and ultimately influences longevity. These findings constitute the basis for the “Hormesis Theory of Aging” [25,26]. Overall, it seems plausible that biological systems tend to accumulate harmful by-products and damage from endogenous or environmental sources over time due to an inherently limited capacity and flexibility of detoxification and adaptive mechanisms. This buildup is influenced by the cell's metabolism, available protective systems and genotype, and may be decisive for the rate of aging [27]. Thus, aging is a complex, multifactorial and highly heterogenous process (between species, individuals of one species or between organs/cells of one organism), and there remains no clear consensus on primary causes. Animal models, including yeast, nematodes, fruit flies, rodents or non-human primates have proven to be instrumental in uncovering key hubs of the aging process [28]. However, translating these findings to humans remains challenging. As a result, the understanding of human aging biology is far from complete. While aging in humans typically occurs over decades, individuals with Hutchinson–Gilford Progeria Syndrome (HGPS) experience accelerated aging and may unlock molecular mechanisms in time lapse.

HGPS is a rare genetic disorder characterized by rapid and premature aging in children [29,30], with an estimated incidence of 1 in 10 million births. Children with HGPS exhibit several signs of premature aging, including alopecia, hearing loss, wrinkled skin, delayed wound healing, osteopenia, joint stiffness, an enlarged skull and lipodystrophy, which can be attributed to problematic adipogenesis [[30], [31], [32], [33]]. They often suffer from progressive atherosclerosis, making heart disease (myocardial infarction or stroke) the leading cause of death. HGPS is caused by a *de novo* point mutation (C1824T) in the *LMNA* gene, encoding lamin A, a key component of the nuclear lamina usually supporting nuclear structure and function. The mutation leads to an alternatively spliced prelamin A protein and the generation of progerin, a truncated and constantly farnesylated lamin A that remains aberrantly anchored to the nuclear envelope and disrupts nuclear architecture. Progerin causes heterochromatin loss, nuclear lobulation, defective DNA repair, mitochondrial dysfunction and disrupted redox homeostasis [32]. The accumulation of progerin is also seen in small amounts in normally aging cells [34,35]. Thus, the dramatically increased progerin accumulation in HGPS may be a blueprint for the normal aging process in high speed and presents a helpful model to better understand underlying mechanisms (Table 1).

**Table 1:** Characteristics of normal aging and progeria.

Characteristics	Physiological Aging	Progeria (HGPS)
**Genetic basis**	Biological process influenced by a multitude of genetic and epigenetic perturbations [1]	Caused by a *de novo* point mutation (C1824T) in the *LMNA* gene, generating protein progerin (truncated lamin A) [32]
**Onset & progression**	Gradual onset over decades, with highly variable progression among individuals [36]	Very early onset (children) with rapid progression of “aged” phenotypes over a few years [29,30]
**Nuclear architecture**	Generally intact nuclear lamina, though nuclear morphological changes accumulate slowly over time [37]	Marked nuclear abnormalities (nuclear blebs, lobulation) due to progerin's persistent farnesylation and mislocalization at the nuclear periphery [32]
**Telomere attrition**	Telomeres shorten gradually with each cell division; rate influenced by oxidative stress, inflammation, and telomerase activity [38]	Accelerated telomere shortening; progerin-induced genomic instability further exacerbates telomere dysfunction [39]
**Redox status**	Gradual increase in reactive oxygen species (ROS) and oxidative damage with age; declining efficiency of antioxidant defenses (e.g., NRF2) in older cells/tissues [40]	Elevated ROS and oxidative stress at a younger age; NRF2 mislocalization/sequestration by progerin impairs the antioxidant response and heightens oxidative damage [41]
**Mitochondrial function**	Mitochondrial dysfunction emerges over time (e.g., decreased ATP generation, increased ROS leakage, poorer quality control), but changes can be partially slowed by lifestyle [42]	Marked mitochondrial defects occur early, linked to progerin-driven nuclear dysregulation, oxidative stress, and inefficient mitophagy [43]
**Proteostasis**	Proteostatic network become less efficient with aging, leading to gradual protein misfolding and aggregation [44]	Pronounced proteostasis imbalance; faster accumulation of misfolded proteins [45]
**Stem cell function**	Progressive decline in stem cell pool, reduced regenerative capacity in tissues (muscle, bone marrow, etc.) over decades [46]	Rapid depletion and dysfunction of mesenchymal stem cells and other stem cell types; osteopenia, lipodystrophy, and poor tissue repair manifest very early in life [47]
**Inflammation**	Chronic, low-grade inflammation gradually builds with age, contributing to tissue damage and disease (heart disease, neurodegeneration, etc.) [48]	Elevated pro-inflammatory signals appear early and potently, accelerating tissue pathology; chronic inflammation fosters atherosclerosis, cardiovascular complications, and accelerated aging features [49]
**Clinical impact**	Health challenges (heart disease, cancer, neurodegeneration etc.) typically show up in later decades [50]	Children with HGPS exhibit alopecia, hearing loss, wrinkled skin, delayed wound healing, osteopenia, joint stiffness, an enlarged skull and lipodystrophy [[30], [31], [32], [33]]

### 1.2 NRF2 and AMPK function and regulation

NRF2 (Nuclear factor E2 p45‐related factor 2) is a leucine zipper transcription factor and a main orchestrator of cell resilience against proteotoxic, xenobiotic and oxidative insults [51]. In the absence of appropriate stressors, NRF2 is synthesized and rapidly degraded by the action of KEAP1 (Kelch-like ECH- associated protein, βTrCP (β-transducin-repeat containing protein) or Hrd1, also called synoviolin, that tag NRF2 with ubiquitin for proteasomal degradation [[52], [53], [54], [55], [56]]. However, in the presence of stressors, ubiquitination of NRF2 ceases, allowing *de novo* synthesized NRF2 to migrate into the nucleus. There, NRF2 activates its target genes, by binding to their antioxidant response elements (ARE) in the promoter or enhancer regions, in concert with members of the small musculo aponeurotic fibrosarcoma (MAF) family which serve as obligatory dimerization partners. By regulating the expression of more than 250 NRF2 target genes, NRF2 impinges among others on redox homeostasis (e.g. *TXN1*, *SRXN1*, *GCLM*, *PRDX1*), detoxification (*NQO1*, *GSTM1*, *GSTA1*, *AR1C1*, *UGT*), lipid and glucose metabolism (*PPARγ*, *LIPH*, *AWAT1, G6PDH, TKT*), heme/iron metabolism (*HMOX1, FTH*), contributing to cytoprotection, stress relief and adaptive homeostasis (reviewed in Refs. [[57], [58], [59]]).

AMP- activated kinase (AMPK) is a trimeric serine/threonine kinase consisting of a catalytic α subunit and regulatory β and γ subunits. Its activity is promoted by a low cellular ATP/AMP ratio, among other cues [60], making it a central energy sensor. [61]. Activated AMPK inhibits anabolic processes that consume ATP, such as protein and lipid synthesis, and promotes catabolic pathways that generate ATP, such as glycolysis and fatty acid oxidation, thus restoring depleted ATP levels. AMPK also regulates mitochondrial biogenesis and autophagy, further helping cells to adapt to energy shortages [61]. Besides energy balance, AMPK is also connected to redox homeostasis: AMPK is directly (by thiol modification) and indirectly (by activation of upstream kinases) activated by elevated ROS levels and contributes to reduced ROS production, e.g. by bolstering mitochondrial number, integrity and function [[62], [63], [64]].

Notably, NRF2 and AMPK signaling also intersect during the cellular stress response: activated AMPK can modulate NRF2 activity either indirectly by regulating a) the GSK3β/βTrCP axis, b) autophagic degradation of KEAP1, c) acetylation of NRF2 via HDAC/HAT activity and in the abundance of acetyl-CoA, or d) directly through phosphorylation, thus linking energy sensing with redox signaling. This synergy has been described in several contexts, including cancer, inflammation, diabetes and neurodegenerative diseases (reviewed in Ref. [65]).

## 2 NRF2 signaling in aging and progeria

### 2.1 NRF2 and aging

Aging is associated with the accumulation of reactive species, damaged biomolecules and declining cell function. NRF2 contributes to preserved cellular redox, metabolic and protein homeostasis and reduced inflammation, which are essential for healthy aging. In addition, numerous studies have reported a reduction of NRF2 levels/activity with age [[66], [67], [68], [69], [70], [71]]. Therefore, its role for aging and lifespan has been investigated in numerous different experimental systems, ranging from *in vitro* cell culture to *in vivo* (small animal) models; selected studies exemplify that (i) depletion of NRF2 boosts aging phenotypes or shortens lifespan, or (ii) increased NRF2 activity (via genetic or pharmacological means) leads to a longer life span or decelerated aging.

Loss or overexpression of SKN-1, the mammalian NRF2 ortholog in *C. elegans*, resulted in shorter or prolonged lifespan, respectively [72]. Naturally long-lived rodents like the naked mole rat possess higher Nrf2 levels, activity and signaling than short-lived mice. Their elevated transcript levels of *Hmox1*, *Gsta1* and *Nqo1* led to a more profound **reduction of oxidative stress** and were positively correlated with maximum lifespan potential (MLSP) [73]. The increased NRF2 levels and activity and MLSP in naked mole rats were associated with lower levels of KEAP1 and βTrCP, the main negative regulators of Nrf2. Additionally, mild upregulation of Nrf2 extended the lifespan of *Drosophila melanogaster* [74]. In other studies it was shown that humans experience a loss or reduced responsiveness of NRF2 during aging, underlining the apparently species-independent association of low NRF2 levels/stress responsiveness, high redox stress and progressive aging [66,70,75].

Closely connected with the imbalanced ROS production is the age-related **mitochondrial impairment,** where mitochondria can be both source and target of reactive species [76,77]. Mitochondria not only are the primary site for production of ATP and key substrates for metabolism, but also markedly participate in intra-organelle signaling and cell fate decisions, underlining their importance for healthy aging. NRF2 activity was positively correlated with mitochondrial turnover (biogenesis and mitophagic clearance ↑) and function (mitochondrial potential ↑, mitochondrial fusion ↑, efficiency of OXPHOS↑ (ROS production ↓, ATP yield ↑) and fatty acid oxidation ↑), as consistently seen in model systems with either NRF2-activation or NRF2-depletion (reviewed in Refs. [[78], [79], [80], [81]]). Accordingly, tomatidine, a compound derived from unripe tomatoes, increased *C. elegans* lifespan by affecting mitochondria dynamics. Specifically, tomatidine induced a mild increase in ROS levels, inducing mitohormesis. As a result, the SKN-1/NRF2 pathway was activated and mitophagy was induced, leading to increased lifespan and improved aging-related muscle function [82,83].

Unmitigated redox stress favors **telomer attrition**, a biomarker of aging. Telomers represent protective ends of eukaryotic chromosomes. Due to low expression of the TERT (telomerase reverse transcriptase) subunit of the telomerase enzyme, telomeres progressively shorten with each cell division due to the inability of the DNA polymerase to synthesize in a 3′–5′ direction and fully replicate the lagging strand. Telomere shortening drives cellular senescence and growth arrest. Due to their repetitive G-rich repeats, telomers are also preferred sites for production of 8-oxoguanine (TelOxidation) [38], which in turn further accelerates telomere shortening and, consequently, leads to premature senescence and genomic instability. Conceivably, NRF2 activity and its impact on the redox network could be associated with TERT expression [[84], [85], [86]]. Furthermore, several pharmacological or food-derived activators of NRF2 signaling, such as dimethyl fumarate or poly-unsaturated fatty acids, have been shown—partially in separate studies, though—to enhance TERT activity, reduce telomere attrition, and mitigate health decline over time [68,87,88].

Besides telomers, proteins, as the most abundant macromolecules of mammalian cells, are especially susceptible to oxidative damage and imminent misfolding or aggregation. Cells can keep their **proteostasis** by protection, repair or turnover. The systems accomplishing the degradation of dysfunctional proteins are the ubiquitin-(20S) proteasomal system (UPS) and the autophagy-lysosomal system, the latter also taking care of damaged organelles, like mitochondria. Intriguingly, (i) many age-related pathologies and the aging process itself are accompanied by a dysregulation of UPS or autophagy [[89], [90], [91], [92], [93], [94]] and (ii) NRF2 transcriptionally activates a plethora of genes involved in proteostasis, including repair enzymes, proteasomal subunits [95,96] and autophagy regulators [97]. Therefore, nature-derived NRF2 activators, including 18alpha-glycyrrhetinic acid, oleuropein, or spermidine, were connected not only to improved proteostasis but also to reduced aging phenotypes [[98], [99], [100], [101]].

Inflammageing refers to the chronic, low-grade, sterile **inflammatory burden** that comes with the accumulation of senescent (immune) cells and/or gut dysbiosis/leakiness during aging [102], constitutes a component of many chronic diseases [103] and adds to a shortened health-span. Activation of NRF2 possesses anti-inflammatory potential and contributes to an intact intestinal barrier, thus potentially alleviating inflammaging and chronic inflammation [67]. Accordingly, *Nrf2*^−/−^ mice exhibited increased neuroinflammation in the MPTP mouse model for Parkinson's disease [104] and tert-butyl-hydroquinone diminishes LPS-induced neuroinflammation and microglia activation in mice via the NRF2/HO1 axis [105].

Multiple aging studies have also unveiled a connection of NRF2 activity with **stem cell function**. The decline in NRF2 expression correlated with an age-related reduction in the regenerative capacity of neural stem progenitor cells (NSPCs) [106]. A comparison between *Nrf2* knock out and *Nrf2* wild type rats of different age indicated that NRF2 dictates the neuronal versus the glial differentiation in the dental gyrus (DG) region of the hippocampus. Introducing NRF2-overexpressing NSPC into the hippocampus improved neurogenesis and cognitive abilities related to the middle-age period, highlighting the role of NRF2 expression for hippocampal neural stem cells aging, likely via improved redox balance [107]. Also mesenchymal stem cells (MSCs) profit from NRF2 overexpression due to better survival and resilience against oxidative stress [108,109]. Conversely, reducing NRF2 expression (via downregulation of the histone acetyl-transferase KAT6A) in human umbilical cord MSCs diminished stem cell marker expression and impeded osteogenesis. NRF2 furthermore impinged on hematopoietic stem cell (HSC) differentiation, quiescence and self-renewal, in a redox-dependent and –independent manner. Loss of NRF2 led to hyperproliferation and expansion of HSCs, compromising their quiescence and self-renewal properties [107]. NRF2 deficiency in intestinal stem cells of *Drosophila melanogaster* elevated ROS levels and age-driven degeneration of the intestinal epithelium [110], and upregulation of NRF2 via the Notch pathway upon administration of traditional Chinese Zuogui Pills (ZGP) preserved oogonial stemness during ovarian aging in rats [111]. This emerging evidence indicates that NRF2 activation may serve as a protective mechanism against the deterioration of various stem cell types by controlling and balancing their self-renewal, quiescence, and regenerative capacities.

**Fig. 1:**
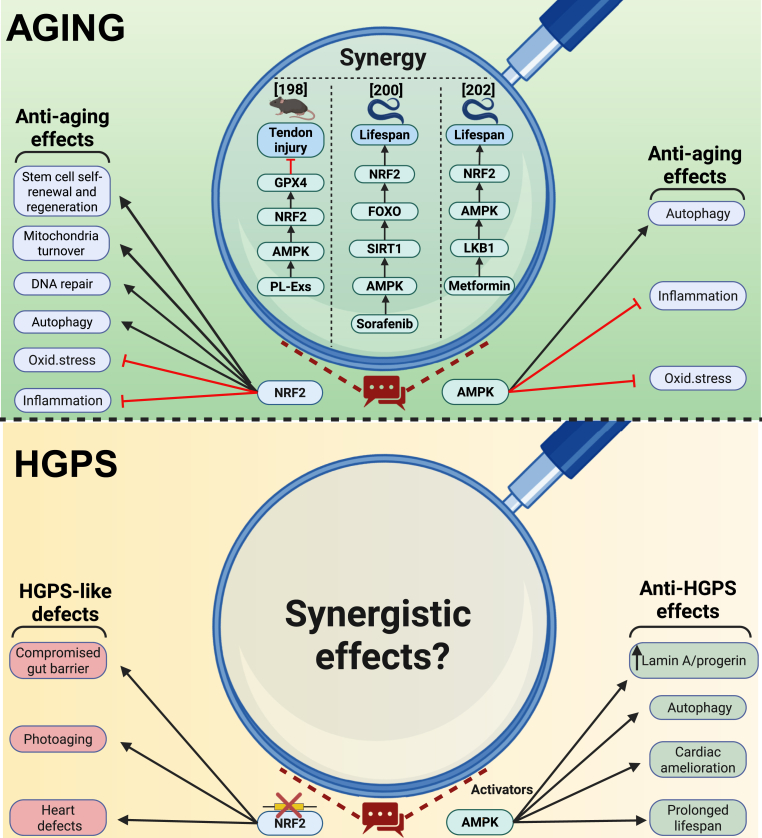
Summary of the main effects of NRF2 and AMPK in different aspects of aging and HGPS. NRF2 and AMPK individually exert multiple effects against aging and Hutchinson Gilford Progeria syndrome (HGPS). Magnifying glasses indicate all current research studies that demonstrate a definite crosstalk between AMPK and NRF2 in anti-aging and anti-HGPS pathways, when applicable.

Overall, there is strong data that declining NRF2 activity underlies diverse hallmarks of aging and its activation favors DNA integrity, survival and differentiation of stem cells, or proteostasis, among others (Fig. 1, top left). Its effects are dependent on or independent of its exerted redox modulation (Table 2). However, the homeostatic network around NRF2 and aging is too complex to allow a binary “good-evil” scheme of thinking. Likewise, overly active (as with constitutively active) Nrf2 signaling and activity of downstream antioxidant enzymes can also exert additional harm on the organism [[112], [113], [114]]. This implies that e.g. both oxidative and reductive stress, depending on nature (reactive species, affected organelles/genes), magnitude, duration, timing, and cellular context, may have partly conflicting impact on organismal health and the aging process.

**Table 2:** Redox-dependent and -independent effects of NRF2.

Hallmark of Aging	Redox-Dependent Effects of NRF2	Redox-Independent Effects of NRF2
**Telomere attrition**	Protection of telomeric DNA from oxidative lesions [115]	Enhanced TERT activity to maintain genomic stability [68,87,88]
**Genome instability**	Preserved DNA integrity by reduced oxidative stress [116]	Induced expression of DNA repair genes [117]
**Cellular senescence**	Reduced ROS-mediated p21 activation and other stress responses leading to senescence [40]	Regulation of expression of senescence-associated genes [118]
**Mitochondrial impairment**	Removal of excess mitochondrial ROS, reducing oxidative damage to mitochondrial membranes and proteins Improved redox balance in the electron transport chain [[78], [79], [80], [81]]	Enhanced mitochondrial biogenesis and mitophagy (through PGC-1α signaling), leading to efficient mitochondrial turnover and energy production [81]
**Disrupted proteostasis**	Preserved protein function by reducing oxidative modifications [51]	Enhanced expression of genes involved in proteostasis such as proteasomal subunits and autophagy-related genes [95,96]
**Inflammation**	Reduced ROS-driven proinflammatory cascades (e.g., NF-κB activation) [119]	Direct suppression of inflammatory gene expression (e.g., IL-1, TNF-α) [119] Preserved gut barrier function (less endotoxin leakage → lower systemic inflammation [67]
**Stem cell exhaustion**	Protection of stem cells (HSCs, MSCs, NSCs) from excess ROS, preserving self-renewal [108,109]	Maintained stemness, quiescence, and proper differentiation signals by controlling transcription factors and epigenetic regulators [120]

### 2.2 Impaired NRF2 function in HGPS

Compelling evidence for NRF2 repression in human aging came from studies on Hutchinson-Gilford progeria syndrome (HGPS), a rare and fatal premature aging disorder. This condition results from the overexpression of a mutant form of Lamin A, known as progerin, which notably sequesters NRF2 and prevents its activation and functionality.

As mentioned, HGPS is a rare genetic disorder with an accelerated aging process, and recent studies suggest that impaired NRF2 activity may contribute significantly to the early onset of cellular degeneration observed in progeria. By employing a cell-based high throughput siRNA screening, Kubben et al. identified the NRF2 antioxidant pathway as a major player in HGPS [121]. Downregulation of NRF2 in wild type fibroblasts decreased the expression of NRF2 target genes and elevated ROS levels to a similar extent as progerin, recapitulating HGPS-related cellular defects. Additionally, overexpression of NRF2 rescued its activity in HGPS mesenchymal stem cells, known for their defective response to oxidative stress. Mechanistically, the mutant form of lamin A, progerin, impedes the transcriptional activity of NRF2 by causing its subnuclear mislocalization and consequently its sequestration away of its target genes. Similarly, in the context of DNA repair, Ghosh et al. showed the interaction of SIRT6 with lamin A as well as SIRT6 mislocalization upon the competition of progerin with lamin A [122]. Interestingly, NRF2 has been shown to form a complex with SIRT6, which is also involved in the activation of NRF2-antioxidant target genes [41]. Therefore, sensitivity to oxidative stress is also observed in HGPS mesenchymal stem cells lacking Sirtuin 6 (SIRT6), possibly via the impediment of NRF2 activation. With these findings, it is tempting to speculate that lamin A-NRF2-SIRT6 facilitates oxidative stress responses. However, these responses are impaired in the case of progerin expressing cells, possibly due to the impediment of NRF2-SIRT6 complexes and their localization at the nuclear membrane at a non-functional state.

Mirroring the loss of adipose tissue and lipid profile of HGPS patients, NRF2-deficient mice show reduced adipogenic differentiation [123], as well as further HGPS-related phenotypes, including accelerated photoaging with the formation of skin wrinkles and loss of skin elasticity [121,124]. In addition, NRF2 is an important player in antioxidant defense against cardiovascular pathologies [125] that are also present in HGPS patients [30], and previous studies highlighted the cardioprotective role of NRF2 by inhibiting NF-kB-mediated inflammatory responses, which are present in HGPS patients. In this line of thought, it is notable that proper NRF2 function contributes to an intact gut barrier. Intestinal permeability, in turn, takes its share in the onset of cardiovascular diseases, a major strain for HGPS patients [[126], [127], [128]].

These findings underline the pivotal role of NRF2 in the aging process and its involvement in multiple aspects of progeria, including dysfunction and premature aging of kidney, heart, skin and MSCs (Fig. 1, bottom left).

## 3 AMPK activity in aging and progeria

The following chapter demonstrates the relevance of AMPK activity in aging and in HGPS.

### 3.1 AMPK and aging

Like NRF2, AMPK activity exhibits a decline with age, suggesting that this downregulation may influence the aging process [[129], [130], [131]]. Conversely, increased AMPK activity was associated with prolonged lifespans across different species [132]. Several recent studies support these phenomena.

Administration of lithocholic acid extended lifespan in *C. elegans, D. melanogaster* and mice in an AMPK-dependent manner [133,134]. Activation of AMPK and improved mitochondrial function contributed to longer life spans in tetramethylpyrazine nitrone-treated nematodes [135]. In *C. elegans*, AMPK activation facilitated nuclear translocation of HLH-30 (a TFEB homologue), enhancing autophagy and promoting healthy aging, as observed after glycerol 3-phosphate phosphatase overexpression [136]. In *Nothobranchius furzeri* activation of AMPK y1, whose expression is reduced in aged individuals, counteracted metabolic quiescence and facilitated healthy aging [137]. In *Drosophila melanogaster*, activation of AMPK α was necessary for lifespan extension following inhibition or knockout of muscle Poly(ADP-ribose) polymerase-1 [138].

The reasons for the disrupted AMPK signaling during the aging process are not completely resolved, but may involve epigenetic or mutational changes in AMPK, LKB1 (an AMPK kinase) or upstream phosphatase expression, chronic inflammation, loss of physical activity or altered hormone or metabolite levels [[139], [140], [141]].

As cellular energy sensor, AMPK is ubiquitously expressed (as trimer composed of different tissue-specific isoforms of the α,β and γ subunits) and orchestrates metabolic pathways to maintain **energy balance** and well-being of the organism. Beyond energy homeostasis, AMPK also affects different signaling pathways involved in **cellular stress resistance, autophagic clearance, inflammation, DNA repair or mitochondrial dynamics**, all with positive impact on healthy aging and longevity [131,[142], [143], [144]]. However and in analogy to NRF2, persistent overactivation of AMPK can have deleterious effects, aggravate pathological damage and finally reduce life/healthspan [145,146].

Probably the most plausible link between AMPK activity and lifespan originated from the association of caloric restriction (reduced calorie intake without malnutrition) or exercise with prolonged life and health spans in different model organisms from fruit fly to (non-) human primates [[147], [148], [149], [150]]. Both interventions are characterized by a reduced ratio of energy intake and energy expenditure and hence AMPK activation. On the cellular level, they could be mainly linked with increased mitochondrial biogenesis and integrity, increased autophagy or reduced oxidative, proteotoxic or inflammatory markers. On the molecular level, consistently recurring themes, besides AMPK, included altered SIRT-, mTOR-, FOXO-, CREB-, IGF 1/insulin or NRF2 signaling. These pathways have in common that they are sensors or executors of nutrient or energy stress, AMPK can act upstream to control their function and/or that they can feed back on the activity of AMPK. Their intricate signaling network shall be briefly exemplified by the interplay between AMPK and SIRT, AMPK and mTOR as well as AMPK and insulin/IGF1:

AMPK and SIRT1 are energy sensors that respond to increases in cellular AMP and NAD + levels, respectively [151]. SIRT1 is a class III protein deacetylase acting on both histones and transcription factors [152,153]. By modulating FoxO, p53, or NF-κB, SIRT1 takes influence on cellular resilience against redox, metabolic, genotoxic and inflammatory stress, finally contributing to a prolonged lifespan [[154], [155], [156]]. AMPK activation increases NAD + levels, stimulating SIRT1 and activating targets like PGC-1 and thus mitochondrial biogenesis [157]. SIRT1 in turn activates LKB1, a key upstream AMPK activator, creating a positive feedback loop [158]. AMPK-enhanced SIRT1 activity also leads to deacetylated autophagy proteins like Atg5, Atg7, and Atg8, preventing the accumulation of damaged organelles by increased autophagy [159].

Together with mTOR, AMPK forms another nutrient/energy sensitive couple that antagonistically regulates autophagy. Under energy stress, activated AMPK is a key inducer of autophagy, which declines with aging [160,161]. This decline leads to waste accumulation, cellular senescence and age-related diseases [162,163], rendering autophagy critical for stress resistance and longevity [164]. AMPK promotes autophagy by inhibiting mTORC1, which is a potent autophagy suppressor activated by growth factors and nutrients. AMPK suppresses mTORC1 activity by phosphorylating Raptor or TSC2 [165]. mTORC1 inhibits autophagy by phosphorylating ULK1, 1 (Unc-51-like kinase 1), a key regulator of autophagosome formation, and hereby preventing ULK1 from initiating autophagy [166]. AMPK counteracts this by phosphorylating ULK1 and dissociating mTORC1 from the ULK1 complex [167,168].

The insulin/IGF signaling pathway and its analogues in different species usually signal high nutrient supply, favor growth and anabolic processes while they reduce longevity through mTOR activation and/or inhibition of autophagy [[169], [170], [171]]. Being part of the somatotropic axis in mammals, a deficient insulin/IGF1 signaling can lead to dwarfism. Intriguingly, dwarf mice, e.g. Ames, Snell and Littlemice, survive their wild type counterparts [172], stressing the pro-aging effect of insulin/IGF-1. In light of its link to AMPK, the insulin/IGF signaling inhibits AMPK activity via downstream activation of AKT, which phosphorylates AMPK at Ser485/Ser491 and impedes activating phosphorylation of AMPK at Thr172 through LKB1 [173]. Conversely, AMPK suppresses insulin/IGF signaling by phosphorylating IRS-1, reducing signal relay from the ligand-bound receptor down to AKT and mTOR [174]. Interestingly and somewhat in contrast to the mutual inhibition of insulin and AMPK signaling, AMPK activation by pharmacological agents such as metformin or by physical exercise enhances insulin sensitivity and glucose uptake [175,176], but still dampens energy consuming processes. Activated AMPK apparently can bolster the metabolic, while hindering the pro-aging effects of the insulin/IGF1 signal. Both pathways are subjected to a complex cross-regulation, with insulin dominating during growth and AMPK critical for stress resilience and autophagy during aging [131].

Overall, AMPK activation phenocopies some aspects of caloric restriction and plays a pivotal role in regulating energy metabolism, autophagic clearance and cellular resilience to oxidative stress and inflammation (Fig. 1, upper right), key factors in extending health- and lifespan. Notably, the positive effect of AMPK on aging-related features is in accordance with the positive impact of NRF2, described above. More studies would be necessary to test whether the two proteins act independently, or if they act in concert on the same pathways (see also chapter 4).

### 3.2 The effect of AMPK activation in HGPS

Previous research highlighted the role of serine-arginine rich factor, SRSF1, in the regulation of LMNA alternative splicing, with its suppression leading to the reduction of progerin mRNA expression and protein levels [177]. Specifically, knocking down *SRSF1* in HGPS-like mouse embryonic fibroblasts downregulated progerin levels [178]. Almost a decade ago, scientists suggested that the activation of AMPK by a plethora of different compounds increased p32, which negatively regulates SRSF1, favoring lamin A production instead of progerin [179]. The anti-diabetic drug metformin, a well-known AMPK activator, decreases the expression of SRSF1 and progerin in a) *Lmna*^*G609G/G609G*^ mouse primary fibroblasts that phenocopied the pathological defects of HGPS and b) in mesenchymal stem cells from HGPS-derived induced pluripotent stem cells, favoring a high lamin A/progerin ratio [180]. In addition, the aforementioned activator improved significantly the premature osteogenic differentiation of HGPS MSC and their nuclear shape organization, as observed in Alkaline phosphatase and immunostaining assays, respectively.

In addition to metformin, a spectrum of additional AMPK activators has been discovered to restore cellular alterations and the pathological defects observed in HGPS cells, mainly by regulating lamin A/progerin ratio in alternative splicing, autophagy, and the upregulation of NRF2 and PGC-1. This spectrum includes sulforaphane, rapamycin, methylene blue, MG132, vitamin D and all-trans retinoic acid [177,[181], [182], [183], [184], [185]]. Taking into consideration that these compounds activate AMPK, and its activation regulates in a positive manner all the aforementioned phenomena and alleviates the pathological HGPS defects, it was suggested that AMPK activation might be the common denominator, connecting all those different compounds with the restoration of HGPS-related cellular changes.

In addition, Chen et al. discovered that isoproterenol impeded LKB1-AMPK interaction and reduced phosphorylation of AMPKα and FOXO3A. This effect weakened the cardiac atrophy phenotype and prolonged lifespan in a progeroid mouse model, mediating enlargement of cardiomyocytes to their former size and restoring cardiac function [186]. In addition, a recent study revealed a connection of AMPK and miR29b2/c, a microRNA expressed in the central nervous system, implicated in the aging process. miR29b2/c KO mice display a progeria like phenotype, with its deficiency resulting in the upregulation of AMPK activity. Elevated AMPK activity in glia seems to exert a protective role in the nigrostriatal pathway in miR29b2/c KO mice [187], underlying once more the important role of AMPK activity in progeria and progeria-phenocopying models.

Interestingly, although AMPK activation alleviates aging-related pathological defects via enhancing autophagy, and promotes lifespan extension, Mariño et al., reported that the LKB1-AMPK-autophagy axis is promoted in the *Zmpste24*^*−/−*^ progeroid mouse model, which bears mutation of a protein mediating lamin maturation. The authors speculated that chronic activation of autophagy in this context can turn its anti-aging mechanism into a pro-aging one [188]. Accordingly, another study, on a direct lamin-mutant genetic background, revealed that overexpression of the secreted proinflammatory cytokine C–C motif chemokine ligand 2 (Ccl2) decreases the lifespan of *Lmna*^G609G/+^ progeroid mice, which show lower levels of total AMPK but higher ratios of pAMPK/AMPK, in comparison to WT mice. In addition, increased autophagy was also reported in the *Lmna*^G609G/+^ progeria mouse model [189]. These results could imply a different role of autophagy in the context of normal aging, which is reversed in the case of accelerated aging in progeria. Alternatively, increased activity of AMPK, leading to increased autophagy in progeria models, could be an attempt to alleviate metabolic stress, but not sufficient to rescue the progeria phenotype. A summary of the main anti-HGPS effects of AMPK is provided in Fig. 1 (bottom right).

## 4 AMPK/NRF2 crosstalk in aging and HGPS

Both AMPK and NRF2 signaling decline with age and increasing their signaling consistently counteracts aging hallmarks. Moreover, cooperativity between the two players can exist on multiple levels: AMPK can modulate NRF2-signaling by a) inhibition of the GSK3β/βTrCP axis of NRF2 degradation, b) the AMPK -enhanced autophagic degradation of KEAP1, or c) direct phosphorylation of NRF2 by AMPK and hence altered stability, nuclear translocation or gene transactivation of NRF2. In reverse, NRF2 can impinge on redox-and energy sensitive AMPK signaling by alteration of the cellular redox state or metabolic program [54,65].

Numerous compounds have been studied as potential geroprotectors, with the goal of extending lifespan and combating aging across various model systems. Many of these compounds, particularly those derived from plants or food sources, have been identified in previous research as influencing both NRF2 and AMPK signaling. However, their potential dualistic basis for geroprotection remains underexplored, as for instance in the case of Aronia melanocarpa polysaccharide [190], 2′-Fucosyllactose [191], resveratrol [192], mulberry leaves [193], Xiyangshen Sanqi Danshen granules [194], green tea polyphenols [195] or ergothioneine [196] (Table 3), where expression levels or activity of AMPK and NRF2 were monitored but not thoroughly questioned for functional cooperativity.

**Table 3:** Summary of the effect of geroprotectors on AMPK and NRF2.

Geroprotector	Model organism	Tissue	Target	Effect on target	Reference
Aronia melanocarpa polysaccharide	*Mus musculus*	Brain	AMPK	↑ levels	[190]
			NRF2	↑ nucleus levels	
2′-Fucosyllactose	*Mus musculus*	Colon	(p)AMPK	↑ levels	[191]
			NRF2	↑ levels	
Resveratrol	*Mus musculus*	Kidney	AMPK	↑ phospho-Thr172/total AMPK ratio	[192]
			NRF2	↑ levels (total & nuclear)	
Mulberry leaves	*Mus musculus, Homo sapiens (L01 cells)*	Liver	AMPK	↑ levels	[193]
			NRF2	↑ levels (total & nuclear)	
Xiyangshen Sanqi Danshen granules	*Mus musculus*	Brain	AMPK	↑ levels	[194]
			NRF2	↑ nuclear/cytosolic NRF2 ratio, **AMPK-dependent**	
Green tea polyphenols	*Caenorhabditis elegans*		AMPK	No lifespan increase by green tea polyphenols upon AMPK or Nrf2 inhibition	[195]
			NRF2		
Ergothioneine	*Mus musculus*	Brain	AMPK	↑ levels	[196]
			NRF2	↑ levels	

This gap makes it still challenging to determine whether simultaneous activation of both AMPK and NRF2 can offer a genuine synergistic or additive benefit for lifespan extension compared to activating only one pathway. Additionally, given the closely interwoven signaling networks governing redox and metabolic balance, it remains unclear whether AMPK and NRF2 signaling can be ever disentangled at all. These questions merit further systematic study.

However, some aging studies have already explicitly focused on a potential interconnected AMPK/NRF2 axis (summarized in Fig. 1, top middle). For instance, in a mouse model of skin aging, overexpression of extracellular superoxide dismutase promoted collagen production via the AMPK/NRF2 axis, resulting in delayed skin aging [197]. In another study, platelet-derived exosomes inhibited senescence of tendon progenitor cells via activation of the AMPK/NRF2/GPX4 axis, which resulted in inhibition of lipid peroxidation and ferroptosis and efficient recovery from tendon injury in rats [198]. Coniferaldehyde prolonged lifespan in *C. elegans* WT, but neither in *skn1* (NRF2) nor *aak2* (AMPK) mutant strains, indicating a pivotal role and interdependence of the two players [199]. Again in *C. elegans*, low doses of sorafenib (1 μM) led to increased lifespan, which was abolished in strains lacking AAK or SKN1. The authors promoted a hierarchical AAF2 (AMPK)/DAF16 (FOXO)/SKN1 (NRF2) signaling axis [200]. Two additional studies involving the antidiabetic blockbuster metformin, known to activate AMPK via mitochondrial complex I inhibition and dropping ATP levels [201], are noteworthy. In one study, metformin extended the health span of *C. elegans* in a manner dependent on both AMPK/AKK2 and NRF2/SKN1 signaling [202]. In another, metformin slowed down neuronal senescence and brain aging in male primates in a clearly NRF2-dependent manner, as demonstrated through experimental NRF2 knockdown and overactivation [203]. The authors argue that the geroprotective effects mediated by NRF2 are independent of metformin's well-established mechanisms involving AMPK activation. However, the inclusion of AMPK knockdown or overactivation experiments would have strengthened this claim and provided greater clarity on the potential relevance of a crosstalk between NRF2 and AMPK in the context of aging.

Similarly to the situation in normal aging, roles for AMPK and NRF2 in progeria have been reported individually. However, there is no study directly evidencing a cooperative AMPK/NRF2 axis in progeria (Fig. 1, bottom middle).

## 5 Conclusion

This review summarizes the influence of AMPK -and NRF2 signaling in natural aging and the genetic premature aging disorder, HGPS (Fig. 1).

NRF2 regulates the transcription of a plethora of detoxification and antioxidant target genes, whereas AMPK is a prime control hub in the cell's energy homeostasis. In line with the observed impaired redox and metabolic imbalance, both NRF2 and AMPK activities decline during aging, and activation of either pathway consistently mitigates several hallmarks of aging across various species. Concerning their relevance as validated targets in deceleration of human aging, it is notable that HGPS patients suffer from deficient NRF2 signaling due to sequestration of NRF2 by progerin, able to explain some of the symptoms of HGPS. However, solid proof, e.g. by symptom rescue on an organismal level by forced NRF2 activity or NRF2 target genes, is still missing. A similar picture of consistent correlation and plausibility, but lacking evidence for causality arises with AMPK activation and prolonged human health or lifespan. Increased physical exercise positively and nutrient overflow negatively influences AMPK activity and health, but there is no clearcut proof for causality yet in humans. The situation may be further complicated as there is data on context and duration-dependent detrimental effects of NRF2 or AMPK activation. Furthermore, while NRF2 and AMPK pathways can interact, their interplay in aging and progeria remains underexplored, with most studies focusing on their individual contributions to these processes.

In several animal models, NRF2 and AMPK have been individually shown to affect a number of common aging- and HGPS- related manifestations, restraining oxidative stress and inflammation and promoting autophagy, mitochondria turnover and cardiac function. In addition, crosstalk between AMPK and NRF2 has already been demonstrated in several non-aging settings, including cancer, diabetes, ischemia and liver disease. Notably, only a handful of studies have explicitly addressed the possible crosstalk between AMPK and NRF2 within the same pathway in the context of aging. Even more strikingly, such studies in HGPS are currently completely absent. For instance, it would be interesting to inhibit AMPK and/or its interaction with NRF2 and test the effects on levels and cellular localization of NRF2, as well as NRF2 responses in aging and progeria systems.

Mechanistically, the central role of mitochondria in aging and progeria has been extensively described. In addition, AMPK and NRF2 have been shown to promote mitochondrial turnover. Therefore, an attractive setting to study the interplay between AMPK and NRF2 in aging/progeria would be mitochondrial dynamics. Overall, there is still a gap in our knowledge about the detailed role of the AMPK/NRF2 axis in aging and progeria, calling for additional systematic analyses. Such efforts could elucidate the possible hierarchical relationship between AMPK and NRF2 in aging and progeria, potentially providing crucial insights in their pathomechanisms and refining therapeutic efforts against progeria and age-related consequences in multiple organs.

Future aging research will benefit from recent advancements in genetic tools, single-cell omics technologies, high-throughput bioenergetics, improved methods for detecting and neutralizing reactive species, and cutting-edge informatics. Integrating metabolism and redox biology with aging phenotypes across complex systems and interconnected tissues may reveal key regulatory nodes driving the aging process over time, further clarifying the roles of AMPK and NRF2 individually or synergistically. This progress, together with the discovery or development of novel specific modulators, may pave the way for a rational anti-aging strategy in humans centered on targeting AMPK and/or NRF2 signaling which at this point seems promising based on their positive effects on cellular stress resistance, proteostasis, inflammation and lifespan in model organisms. On the other hand, caution should be exercised with AMPK/NRF2-activating approaches, considering the adverse effects reported on NRF2 and AMPK overactivation. Specifically, overactivation of the NRF2 pathway has been reported in several cancers, including non-small cell lung cancer, hepatocarcinoma, multiple myeloma and head and neck carcinoma, among others, providing protection of tumor cells against oxidative stress [204,205]. Accordingly, AMPK overactivation had deleterious consequences in the contexts of ischemic stroke and myocardial ischemia [145,146] and induced obesity and hepatic steatosis in a mouse model [206].

In conclusion, while AMPK and NRF2 activation represents a promising anti-aging strategy due to their broad roles in enhancing cellular stress resistance, autophagy, mitochondrial turnover and metabolic homeostasis, a nuanced approach is essential to avoid potential adverse effects linked to their overactivation. Future interventions should aim for context-specific, temporally controlled, and dose-optimized modulation. In this regard, emerging gene modulation technologies such as CRISPR activation (CRISPRa) [207,208] offer an exciting avenue for precise, reversible, and tissue-specific upregulation of endogenous AMPK and NRF2 pathways without permanent genomic alterations. Although still under preclinical investigation, CRISPRa could enable safer and more targeted interventions in human aging by aligning gene activation with individual biological needs and minimizing systemic side effects. Such strategies may help harness the beneficial effects of AMPK and NRF2 while minimizing the risks, paving the way for rational and safe anti-aging therapies in humans.

## CRediT authorship contribution statement

**Eleni Petsouki:** Writing – review & editing, Writing – original draft, Conceptualization. **Vasileios Gerakopoulos:** Writing – review & editing. **Despoina D. Gianniou:** Writing – review & editing. **Elke H. Heiss:** Writing – review & editing, Writing – original draft, Funding acquisition. **Ioannis P. Trougakos:** Writing – review & editing.

## Declaration of competing interest

The authors declare that they have no known competing financial interests or personal relationships that could have appeared to influence the work reported in this paper.

## Data Availability

No data was used for the research described in the article.
